# Supplementary material for: MetaRibo-Seq measures translation in microbiomes
Source: Nat Commun. 2020 Jun 29;11:3268. doi: 10.1038/s41467-020-17081-z (PMC7324362; doi:10.1038/s41467-020-17081-z)
Supplement: Supplementary file 10 — Supplementary Data 7 [file 41467_2020_17081_MOESM10_ESM.zip › File2/Confidence_High_Taxonomy/58111_out.krona.html]

Javascript must be enabled to view this page.

members
magnitude
magnitudeUnassigned
count
unassigned
taxon
rank

58111\_out

27

superkingdom
27
2

27
phylum
1239

class
27
186801

order
27
186802

541000
2
family

2
genus
1263

657323
species
1

SRS147139\_contig\_number\_37074

species
1
2293189

SRS023914\_contig\_number\_contig-100\_38.164684

186803
family
25


SRS1055043\_contig\_number\_360
658089
1
species

572511
24
genus


SRS018351\_contig\_number\_3194SRS019445\_contig\_number\_37425SRS144183\_contig\_number\_27724
765821
3
species

33038
species
19

SRS012273\_contig\_number\_48730SRS014948\_contig\_number\_9754SRS015431\_contig\_number\_83824SRS016495\_contig\_number\_15384SRS017521\_contig\_number\_25185SRS019068\_contig\_number\_107622SRS019397\_contig\_number\_13289SRS042284\_contig\_number\_6841SRS050422\_contig\_number\_21434SRS057717\_contig\_number\_7715SRS063040\_contig\_number\_49423SRS077454\_contig\_number\_16270SRS077502\_contig\_number\_19351SRS097889\_contig\_number\_41933SRS142503\_contig\_number\_47873SRS143780\_contig\_number\_32583SRS148721\_contig\_number\_822SRS893259\_contig\_number\_5138SRS893378\_contig\_number\_22445

40520
species
2

SRS016267\_contig\_number\_5744SRS018427\_contig\_number\_27532
